# Evolutionary Insights from a Genetically Divergent Hantavirus Harbored by the European Common Mole (*Talpa europaea*)

**DOI:** 10.1371/journal.pone.0006149

**Published:** 2009-07-07

**Authors:** Hae Ji Kang, Shannon N. Bennett, Laarni Sumibcay, Satoru Arai, Andrew G. Hope, Gabor Mocz, Jin-Won Song, Joseph A. Cook, Richard Yanagihara

**Affiliations:** 1 Pacific Center for Emerging Infectious Diseases Research, John A. Burns School of Medicine, University of Hawaii at Manoa, Honolulu, Hawaii, United States of America; 2 Department of Microbiology, College of Medicine, Institute for Viral Diseases and Bank for Pathogenic Viruses, Korea University, Seoul, Korea; 3 Infectious Disease Surveillance Center, National Institute of Infectious Diseases, Tokyo, Japan; 4 Department of Biology and Museum of Southwestern Biology, University of New Mexico, Albuquerque, New Mexico, United States of America; 5 Pacific Biosciences Research Center, University of Hawaii at Manoa, Honolulu, Hawaii, United States of America; Institute of Molecular and Cell Biology, Singapore

## Abstract

**Background:**

The discovery of genetically distinct hantaviruses in shrews (Order Soricomorpha, Family Soricidae) from widely separated geographic regions challenges the hypothesis that rodents (Order Rodentia, Family Muridae and Cricetidae) are the primordial reservoir hosts of hantaviruses and also predicts that other soricomorphs harbor hantaviruses. Recently, novel hantavirus genomes have been detected in moles of the Family Talpidae, including the Japanese shrew mole (*Urotrichus talpoides*) and American shrew mole (*Neurotrichus gibbsii*). We present new insights into the evolutionary history of hantaviruses gained from a highly divergent hantavirus, designated Nova virus (NVAV), identified in the European common mole (*Talpa europaea*) captured in Hungary.

**Methodology/Principal Findings:**

Pair-wise alignment and comparison of the full-length S- and L-genomic segments indicated moderately low sequence similarity of 54–65% and 46–63% at the nucleotide and amino acid levels, respectively, between NVAV and representative rodent- and soricid-borne hantaviruses. Despite the high degree of sequence divergence, the predicted secondary structure of the NVAV nucleocapsid protein exhibited the characteristic coiled-coil domains at the amino-terminal end, and the L-segment motifs, typically found in hantaviruses, were well conserved. Phylogenetic analyses, using maximum-likelihood and Bayesian methods, showed that NVAV formed a distinct clade that was evolutionarily distant from all other hantaviruses.

**Conclusions:**

Newly identified hantaviruses harbored by shrews and moles support long-standing virus-host relationships and suggest that ancestral soricomorphs, rather than rodents, may have been the early or original mammalian hosts.

## Introduction

Hantaviruses belong to the Genus *Hantavirus* of the Family Bunyaviridae, which includes four other genera (*Orthobunyavirus*, *Phlebovirus*, *Nairovirus* and *Tospovirus*). All members of this large virus family possess a negative-sense, single-stranded tripartite RNA genome, consisting of large (L), medium (M) and small (S) segments, which encode an RNA-dependent RNA polymerase, two envelope glycoproteins (Gn and Gc) and a nucleocapsid (N) protein, respectively [1]–[4]. Insects and arthropods characteristically serve as vectors of viruses in the Bunyaviridae, except for hantaviruses, which instead are harbored by rodents (Order Rodentia, Family Muridae and Cricetidae) [5], [6]. Although not all hantaviruses are pathogenic, several harbored by murine and arvicoline rodents cause hemorrhagic fever with renal syndrome (HFRS) in Eurasia [7], [8], while others carried by neotomine and sigmodontine rodents cause hantavirus cardiopulmonary syndrome (HCPS) in the Americas [9], [10].

Hantavirus enzootics are maintained in multiple rodent species [5]–[8]. However, previous epizootiological studies of small mammals, captured in HFRS-endemic regions, have suggested that shrews (Order Soricomorpha, Family Soricidae) and moles (Family Talpidae) might also serve as reservoir hosts of hantaviruses [11]–[13]. Guided by these reports, we launched an opportunistic search for soricomorph-associated hantaviruses by analyzing archival tissue collections. We thought that the availability of the full genomes of Thottapalayam virus (TPMV) and Imjin virus (MJNV), two prototypic crocidurine shrew-borne hantaviruses, harbored by the Asian house shrew (*Suncus murinus*) [14]–[17] and Ussuri white-toothed shrew (*Crocidura lasiura*) [18], respectively, would facilitate the task of identifying hantaviruses in other soricomorph species. However, the unexpectedly high genetic diversity of soricomorph-borne hantaviruses made designing suitable oligonucleotide primers difficult. We finally succeeded in detecting novel hantaviruses by reverse transcription-polymerase chain reaction (RT-PCR) in tissues of several soricid species, including the Eurasian common shrew (*Sorex araneus*) from Switzerland [19], Chinese mole shrew (*Anourosorex squamipes*) from Vietnam [20], and northern short-tailed shrew (*Blarina brevicauda*) [21], dusky shrew (*Sorex monticolus*) [22] and masked shrew (*Sorex cinereus*) [22] from the United States. Less well-characterized hantaviruses have also been identified in the Therese's shrew (*Crocidura theresae*) from Guinea [23], as well as in the Asian lesser white-toothed shrew (*Crocidura shantungensis*) from Korea, vagrant shrew (*Sorex vagrans*), Trowbridge's shrew (*Sorex trowbridgii*) and American water shrew (*Sorex palustris*) from the United States, and flat-skulled shrew (*Sorex roboratus*) and Laxmann's shrew (*Sorex caecutiens*) from Russia (H.J. Kang, S. Arai and R. Yanagihara, unpublished observations).

The discovery of genetically distinct hantaviruses in soricine and crocidurine shrews, captured in widely separated geographical regions spanning four continents, does not detract from the vast literature on disease-causing rodent-borne hantaviruses. Rather, the demonstration of phylogenetically distinct soricomorph-associated hantaviruses enriches our understanding about their possible origins and suggests that their evolutionary record is far more complex and probably more ancient than originally contemplated. Mounting evidence also suggests that host-switching events among sympatric and syntopic reservoir rodent host species have figured prominently during the distant past and continue to influence the evolutionary dynamics of hantaviruses [24]–[26]. Cross-species transmission, occurring independently in the Old World and New World, also appears to account for the emergence of Asama virus (ASAV) in the Japanese shrew mole (*Urotrichus talpoides*) [27] and Oxbow virus (OXBV) in the American shrew mole (*Neurotrichus gibbsii*) [28]. Recently, the entire notion of co-divergence has been opposed in favor of preferential host switching and local host-specific adaptation [29]. More likely, however, both co-divergence and cross-species transmission have been operative. In this report, a highly divergent hantavirus, designated Nova virus (NVAV), harbored by the European common mole (*Talpa europaea*), provides support for a long-standing virus-host relationship and suggests the possibility that ancestral soricomorphs, rather than rodents, may have served as the early or original mammalian hosts of primordial hantaviruses.

## Materials and Methods

### Tissues

**Figure 1:**
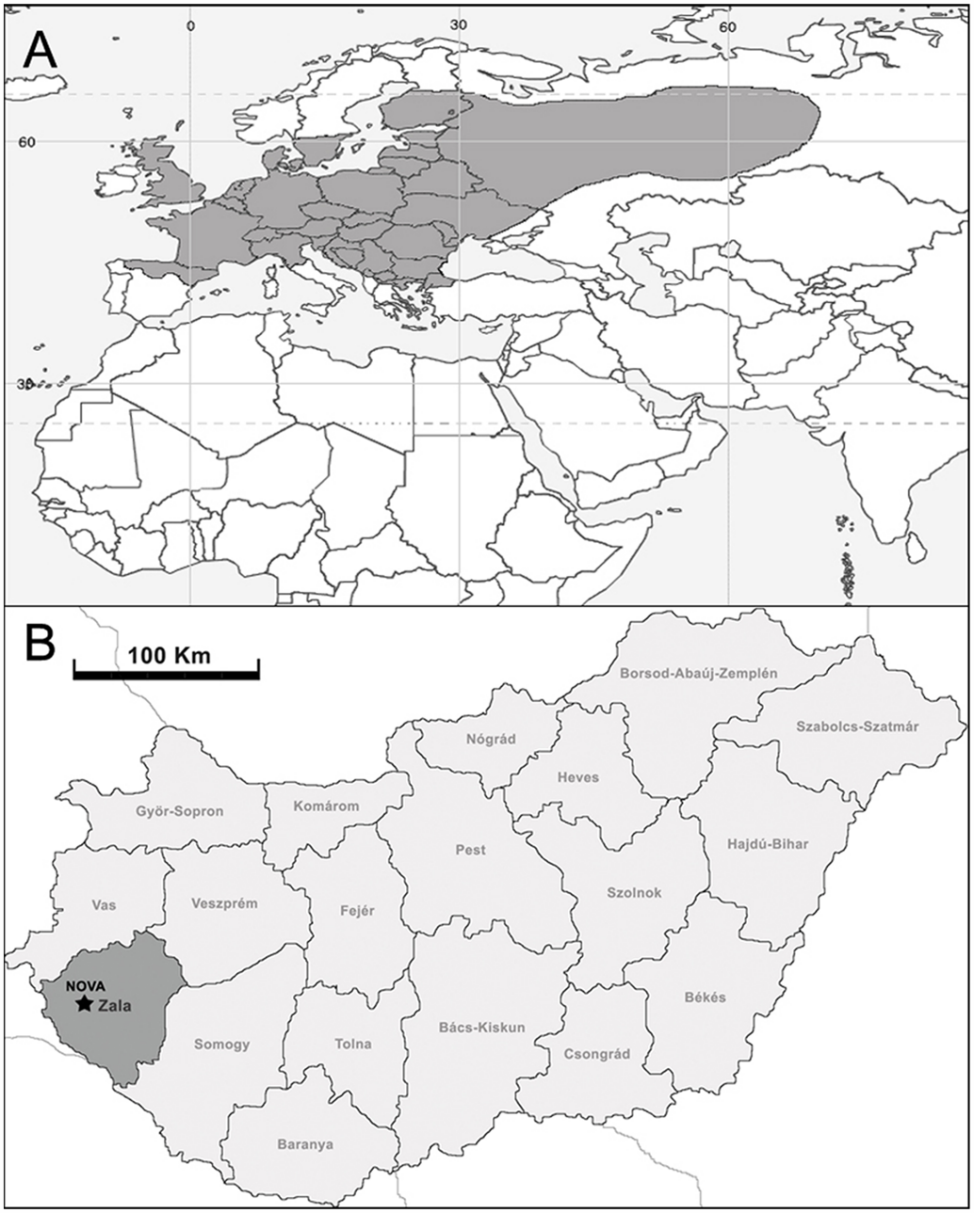
Map, showing the (A) distribution of the European common mole (*Talpa europaea*) in western Eurasia and (B) capture site of the host specimen of Nova virus in Hungary. European common moles are distributed throughout temperate Europe and western Russia. Nova virus RNAs were amplified from liver tissue of a European common mole, captured near Nova (star), in Zala County, Hungary, in July 1999.

Frozen liver or heart and kidney tissues from a convenience sample, consisting of five Chinese shrew mole (*Uropsilus soricipes*), two coast mole (*Scapanus orarius schefferi*), two Townsend's mole (*Scapanus townsendii*), two broad-footed mole (*Scapanus latimanus caurinus*), two long-tailed mole (*Scaptonyx fusicaudus*) and six European common mole (*Talpa europaea*), archived in the Museum of Southwestern Biology at the University of New Mexico in Albuquerque, were studied. European common moles, which are among the most widespread small mammal species in Europe (Figure 1A), were captured in Hungary in 1997, 1999 and 2000, while the other mole species were captured in the United States and China in 1984 and 2005, respectively.

### RNA Extraction and cDNA Synthesis

Total RNA, extracted from liver, heart and kidney tissues, using the PureLink Micro-to-Midi total RNA purification kit (Invitrogen, San Diego, CA), was reverse transcribed, using the SuperScript III First-Strand Synthesis System (Invitrogen, San Diego, CA, USA) and oligonucleotide primer (OSM55, 5′-TAGTAGTAGACTCC-3′) designed from the conserved 5′-end of the S, M and L segments of hantaviruses.

### RT-PCR and DNA Sequencing

Initially, S- and L-segment sequences of rodent-borne hantaviruses, available in GenBank, and our growing sequence database of shrew-borne hantaviruses were aligned using ClustalW [30], and regions exhibiting high homology or sequence conservation were selected for designing oligonucleotide primers in nested RT-PCR. Numerous attempts were required to assess the suitability of the presumptive primers. After the initial detection of hantavirus RNAs, amplification of the full-length S- and L-genomic segments was accomplished using oligonucleotide primers based on newly acquired sequences. Oligonucleotide primer sequences have been deposited as Supplemental Data S1 with the journal.

First- and second-round PCR were performed in 20-µL reaction mixtures, containing 250 µM dNTP, 2 mM MgCl_2_, 1 U of AmpliTaq polymerase (Roche, Basel, Switzerland) and 0.25 µM of each primer. Initial denaturation at 94°C for 5 min was followed by two cycles each of denaturation at 94°C for 40 sec, two-degree step-down annealing from 48°C to 38°C for 40 sec, and elongation at 72°C for 1 min, then 32 cycles of denaturation at 94°C for 40 sec, annealing at 42°C for 40 sec, and elongation at 72°C for 1 min, in a GeneAmp PCR 9700 thermal cycler (Perkin-Elmer, Waltham, MA). Amplicons were separated by electrophoresis on 1.5% agarose gels and purified using the QIAQuick Gel Extraction Kit (Qiagen, Hilden, Germany). DNA was sequenced directly using an ABI Prism 377XL Genetic Analyzer (Applied Biosystems, Foster City, CA).

### Genetic Analysis

Full-length S- and L-segment nucleotide and amino acid sequences of the new hantavirus, amplified from the European common mole, were aligned with publicly available hantavirus sequences, using the ClustalW method (Lasergene program version 5, DNASTAR, Inc., Madison, WI) [30]. Pair-wise comparisons were performed to ascertain the degree of sequence homology. In addition, analysis of the coding regions of the full-length S- and L-segment sequences of NVAV was performed using multiple recombination-detection methods, including HYPHY's Single Recombinant Breakpoint [31], GENECONV [32], Bootscan [33], Chimaera [34], MaxChi [34], 3SEQ [35], RDP [36] and SiScan [37]. The GenBank accession numbers for the NVAV S and L segments are FJ539168 and FJ593498, respectively.

### Protein Analysis and Prediction

For N protein secondary structure prediction, the entire amino acid sequences were submitted to the NPS@ structure server [38]. COILS [39] was also used to scan the N protein for coiled-coil regions. To improve accuracy and to ensure the validity of the predicted structure, five methods - DSC [40], HNN [40], MLRC [41], PHD [42] and PREDATOR [43] - were employed jointly. The minimum number of conformational states was set at four (helix, sheet, turn and coil) for each analysis, and the results were combined into a consensus structure where the most prevalent predicted conformational state was reported for each residue. Color-coded consensus structures for the individual sequences were displayed graphically using a program written in-house, and the outputs were combined into a composite image.

### Phylogenetic Analysis

Phylogenetic trees were generated using the maximum-likelihood (ML) method implemented in PAUP* (Phylogenetic Analysis Using Parsimony, 4.0b10) [44], RAxML Blackbox web-server [45] and MrBayes 3.1 [46]. The optimal evolutionary model was estimated as the GTR+I+Γ model of evolution, as selected by using jModelTest version 0.1 [47]. ML topologies were evaluated by bootstrap analysis of 1,000 neighbor-joining iterations or 100 ML iterations, implemented in PAUP* or RAxML web server, respectively. Bayesian analysis under MrBayes 3.1 consisted of at least two million Markov Chain Monte Carlo generations to ensure convergence across two runs of four chains each, with average standard deviations of split frequencies less than 0.01 and effective sample sizes well over 100, resulting in consensus trees supported by posterior-node probabilities. With a robust phylogeny of shrew- and rodent-borne hantaviruses, we readdressed the co-evolutionary relationship between hantaviruses and their hosts that formed the basis of our predictive paradigm for hantavirus discovery, by comparing the degree of concordance between reservoir host and hantavirus cladograms in TreeMap 2.0b [48]–[50].

### mtDNA Sequencing and Host Phylogeny

To verify the identity of the hantavirus-infected European common mole and to study its phylogenetic relationship, genomic DNA was extracted from frozen liver tissue using the QIAamp DNA Mini Kit (Qiagen), according to the manufacturer's instructions. The entire 1,140-nucleotide region of the cytochrome *b* gene of mitochondrial DNA (mtDNA) was amplified by PCR, using well-tested primers (forward: 5′-CGAAGCTTGATATGAAAAACCATCGTTG-3′; and reverse: 5′-CTGGTTTACAAGACCAGAGTAAT-3′) [51]. PCR was performed in 50-µL reaction mixtures, containing 200 µM dNTP and 1 U of AmpliTaq polymerase (Roche, Basel, Switzerland). Cycling conditions consisted of 8 cycles at 94°C for 40 sec, 55°C for 40 sec and 72°C for 1 min, followed by 30 cycles at 94°C for 40 sec, 50°C for 40 sec and 72°C for 1 min, and 1 cycle at 72°C for 7 min. Amplified DNA was purified and then submitted for automated fluorescent sequencing. Host phylogenies based on mtDNA cytochrome *b* sequences, along with published sequences for shrews and moles for this gene region, were generated, as described previously [21], [22], [27], [28]. Briefly, the ML method and MrBayes 3.1 were used to produce phylogenetic trees.

## Results

### Screening of Mole Tissues

RT-PCR analysis, using oligonucleotide primers targeting the S- and L-genomic segments of hantaviruses, revealed novel hantavirus RNAs in liver and in heart and kidney tissues of a European common mole, captured 4 km northwest of Nova (46°41′2 N, 16°41′2 E), in Zala County, Hungary, in July 1999 (Figure 1B). Unfortunately, lung or other tissues were unavailable from this animal. Tissues from all other mole species were repeatedly negative.

### Genetic Analysis

The entire S- and L-genomic segments of NVAV (strain MSB95703) were amplified from tissues of a European common mole from Hungary. However, multiple attempts to amplify the M segment of NVAV were unsuccessful.

The full-length 1,839-nucleotide S-genomic segment of NVAV contained a single open reading frame (ORF), encoding a 428-amino acid N protein (nucleotide positions, 53 to 1,339), and an approximately 500-nucleotide 3′-noncoding region. Like Hantaan virus (HTNV) and other hantaviruses harbored by murine rodents, as well as two recently described hantaviruses in shrew moles, the NVAV S segment did not have the hypothetical NSs ORF, typically found in hantaviruses carried by arvicoline, neotomine and sigmodontine rodents.

**Figure 2:**
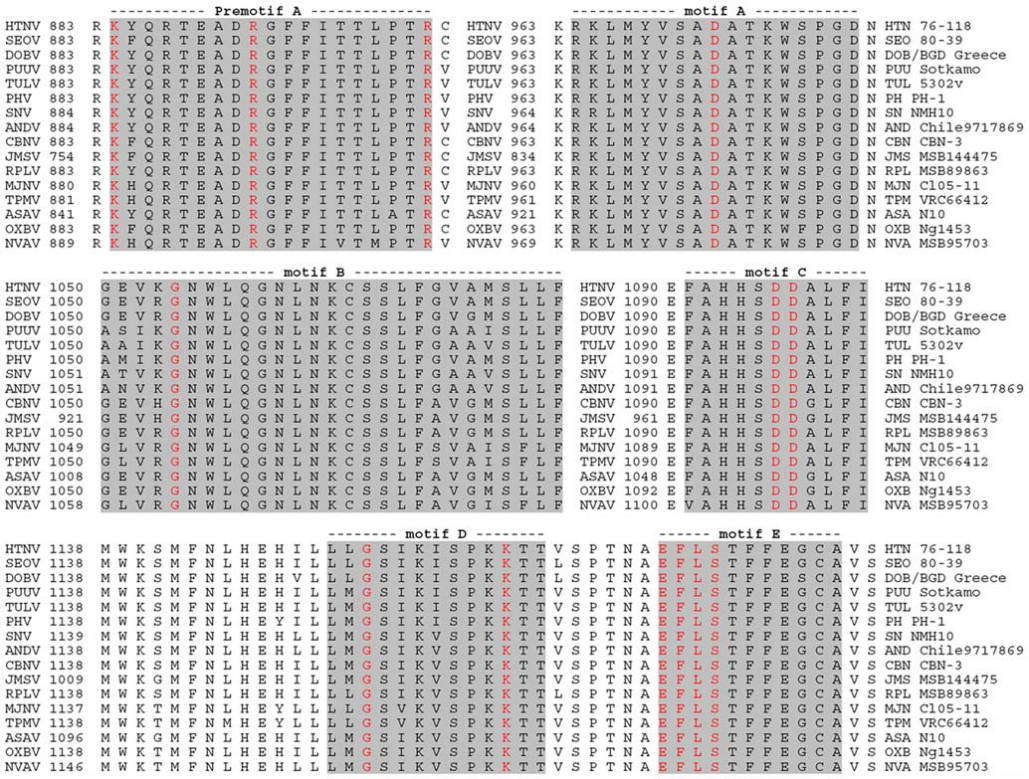
L-segment amino acid sequence alignment of the region spanning the premotif A and motifs A–E in hantaviruses. Highly conserved amino acids are highlighted in red and only presented in polymerases of negative stranded RNA viruses. A multiple amino acid sequence alignment of RNA-dependent RNA polymerases was created using the ClustalW method for the following hantaviruses: Hantaan virus (HTN 76–118, NC_005222), Dobrava/Belgrade virus (DOB/BGD Greece, NC_005235), Seoul virus (SEO 80–39, NC_005238), Tula virus (TUL 5302v, NC_005226), Puumala virus (PUU Sotkamo, NC_005225), Prospect Hill virus (PH PH-1, EF646763), Sin Nombre virus (SN NMH10, NC_005217), Andes virus (AND Chile9717869, AF291704), Thottapalayam virus (TPM VRC66412, EU001330), Imjin virus (MJN Cl05–11, EF641806), Cao Bang virus (CBN CBN-3, EF543525), Jemez Springs virus (JMS MSB144475, FJ593501), Camp Ripley virus (RPL MSB89863, EF540771), Asama virus (ASA N10, EU929078), Oxbow virus (OXB Ng1453, FJ593497) and Nova virus (NVA MSB95703, FJ593498).

The 6,474-nucleotide L-genomic segment of NVAV encoded a predicted RNA-dependent RNA polymerase of 2,157 amino acids. Six major conserved motifs (designated as premotif A and motifs A, B, C, D and E) for RNA virus polymerases, which are shared by all hantaviruses, were also found in NVAV (Figure 2). Premotif A had a conserved lysine and two arginine residues. Motifs A, B and D had conserved aspartate, glycine and lysine, respectively. In motif C, there were two conserved aspartic acid residues. The XDD motif, essential for catalytic activity, and motif E, containing the E(F/Y)XS site, were also present.

When compared with full-length S-segment sequences of other rodent- and soricomorph-borne hantaviruses, NVAV showed moderately low sequence homology, ranging from approximately 54–58% (54–60% in the coding region) at the nucleotide level and 46–53% at the amino acid level (Table 1). Higher amino acid sequence similarities of 58–74% were found in the RNA-binding region than in the overall S segment. Sequence homology was also higher for the L-genomic segment (60–64% and 59–63% at the nucleotide and amino acid levels, respectively) (Table 1). Although there was greater sequence conservation in the L- than S-genomic segment, NVAV represented the most genetically divergent of all previously sequenced hantaviruses, irrespective of host species and geographic origin.

**Table 1:** Sequence similarities (%) of the coding regions of the full-length S and L segments of NVAV strain MSB95703 and representative rodent- and soricomorph-borne hantaviruses.

Virus strain	S segment		L segment	
	1287 nt	428 aa	6474 nt	2032 aa
HTNV 76–118	56.3	51.2	63.1	62.2
SEOV 80–39	55.6	48.6	63.5	62.2
SOOV SOO-1	57.0	50.7	62.8	62.4
DOBV/BGDV Greece	57.1	50.7	63.7	63.0
PUUV Sotkamo	59.5	53.3	63.9	61.9
TULV 5302v	57.4	50.7	63.9	62.1
PHV PH-1	57.9	50.5	62.1	61.7
SNV NMH10	58.0	51.5	64.0	62.2
ANDV Chile9717869	57.3	51.5	63.1	62.0
ARRV MSB73418	57.6	48.0	60.4	59.2
CBNV CBN-3	58.1	50.4	63.8	62.6
JMSV MSB144475	57.0	49.1	62.3	61.7
SWSV mp70	56.3	50.7	59.6	58.7
RPLV MSB89863*	-	-	63.8	62.4
MJNV Cl05-11	53.7	45.6	64.0	63.4
TPMV VRC66412	53.6	46.8	64.5	63.0
ASAV N10	55.4	48.6	63.8	62.4
OXBV Ng1453	57.8	49.9	60.7	60.4

Abbreviations: ANDV, Andes virus; ARRV, Ash River virus; ASAV, Asama virus; CBNV, Cao Bang virus; DOBV/BGDV, Dobrava/Belgrade virus; HTNV, Hantaan virus; JMSV, Jemez Spring virus; NVAV, Nova virus; MJNV, Imjin virus; OXBV, Oxbow virus; PHV, Prospect Hill virus; PUUV, Puumala virus; RPLV, Camp Ripley virus; SEOV, Seoul virus; SNV, Sin Nombre virus; SOOV, Soochong virus; SWSV, Seewis virus; TPMV, Thottapalayam virus; TULV, Tula virus. nt, nucleotides; aa,amino acids.

* The 330-nucleotide region of the RPLV S segment was 45.5% and 39.1% similar to NVAV at the nucleotide and amino acid levels.

Consistent evidence of recombination was not found in the coding regions of the full-length S and L segments of NVAV. That is, while separate regions of potential recombination were found in a few instances, there was poor concordance between the detection methods, making untenable any claims for recombination.

### Secondary Structure of N Protein

**Figure 3:**
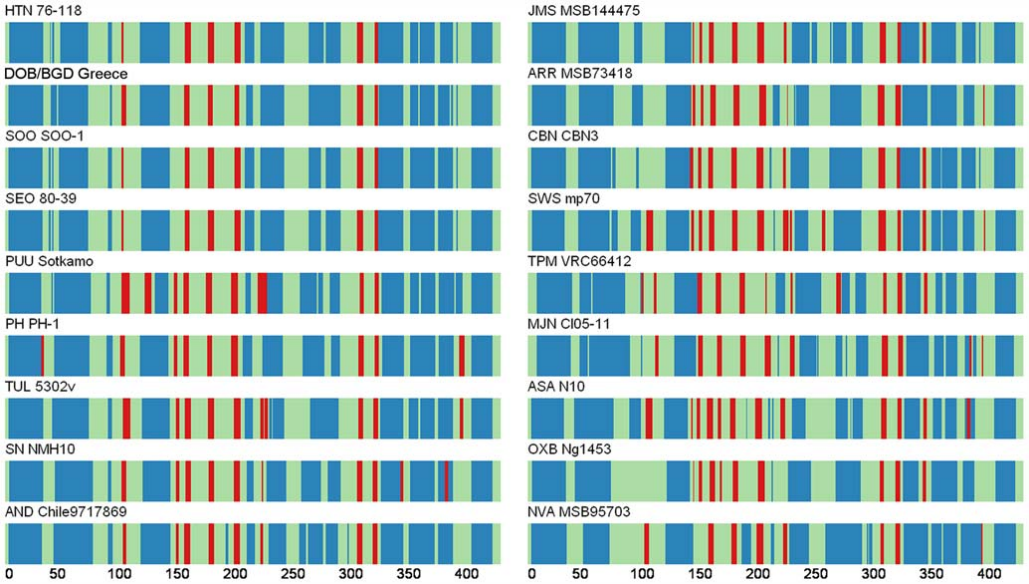
Comparison of consensus secondary structures of entire N proteins of NVA MSB95703 and representative hantaviruses, predicted using several methods, including HNN [20], DSC [26], PHD [42], PREDATOR [16] and MLRC [18] at the NPS@ structure server [12]. N protein structures are shown for rodent-borne hantaviruses (left panel: HTN 76–118, DOB/BGD Greece, SOO SOO-1, SEO 80-39, PH PH-1, PUU Sotkamo, TUL 5302v, SN NMH10 and AND Chile 9717869) and for hantaviruses harbored by shrews (right panel: JMS MSB144475, ARR MSB73418, CBN CBN-3, SWS mp70, TPM VRC66412, MJN Cl05-11) and moles (right panel: ASA N10, OXB Ng1453, NVA MSB95703). Blue bars represent a-helices, red bars β-strands, and green and orange indicate random coil and unclassified structures, respectively.

Despite the relatively low amino acid sequence similarity, the overall predicted secondary structure of the NVAV N protein resembled the N protein structures observed for other rodent-, soricid- and talpid-borne hantaviruses, in that the N protein was comprised of two major α-helical domains packed against a central β-pleated sheet (Figure 3). Multiple sequence alignments (ClustalW) and the COILS coiled-coil prediction method identified two heptad repeats between residues 1 and 63 in NVAV. While the first main α-helix appeared essentially invariant, the second main α-helix had a shorter span than the span of the equivalent α-helices in other N proteins in the representative set. Also, the third larger α-helix was not present in the NVAV N protein, which resulted in an extended loop between the second and fourth α-helices. The absence of the third α-helix was also observed in OXBV Ng1453 and TPMV VRC66412. In the latter N protein, the amino-terminus of the second main α-helix also appeared segmented. The helical structure in the rest of the NVAV N protein appeared less segmented than in most other N proteins and several α-helices were significantly longer in the fold. The extended α-helices imparted stronger helical character and limited the intra-domain flexibility of the molecule.

The central β-pleated sheet motif of NVAV most resembled that of murine rodent-associated hantaviruses, including HTNV 76–118, Dobrava/Belgrade virus (DOBV/BGDV) Greece, Soochong virus (SOOV) SOO-1 and Seoul virus (SEOV) 80-39 (Figure 3). These N proteins had fewer strands than most N proteins in the comparative set. The RNA-binding region, at amino acid positions 175 to 217, showed predominantly β-strands in the NVAV N protein, as for other hantaviruses. However, the NVAV N protein also had a prominent α-helix at residues 184 to 193. Similarly, rodent- and shrew mole-borne hantaviruses, namely Andes virus (ANDV) Chile9717869 and ASAV N10, respectively, exhibited the same helical element in this region, although these α-helices appeared much less well-formed. The distinctive α-helix motif between two β-strands of the RNA-binding region may have a significant effect on binding specificity, especially for NVAV.

### Phylogenetic Analysis

**Figure 4:**
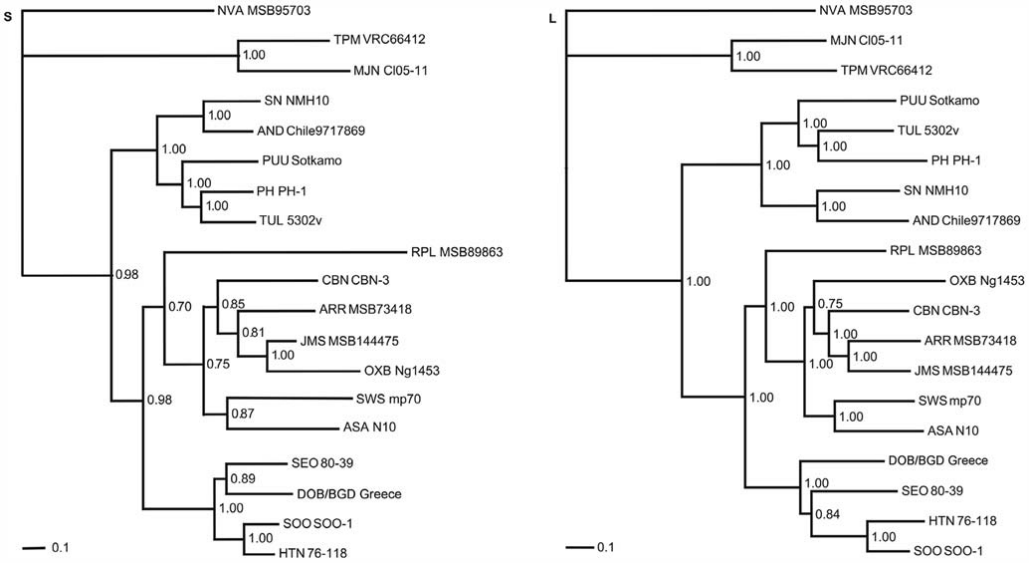
Phylogenetic trees generated by the maximum-likelihood (ML) method, using the GTR+I+Γ model of evolution as estimated, based on the coding regions of the full-length (S) 1,287-nucleotide S- and (L) 6,474-nucleotide L-genomic segments of NVAV. The phylogenetic position of NVA MSB95703 is shown in relationship to representative murine rodent-borne hantaviruses, including HTN 76–118 (NC_005218, NC_005222), SOO SOO-1 (AY675349, DQ056292), DOB/BGD Greece (NC_005233, NC_005235) and SEO 80–39 (NC_005236, NC_005238); arvicoline rodent-borne hantaviruses, including TUL 5302v (NC_005227, NC_005226), PUU Sotkamo (NC_005224, NC_005225) and PH PH-1 (Z49098, EF646763); a neotomine rodent-borne hantavirus, SN NMH10 (NC_005216, NC_005217); and a sigmodontine rodent-borne hantavirus, AND Chile9717869 (NC_003466, NC_003468). Also shown are TPM VRC66412 (AY526097, EU001330) from the Asian house shrew (*Suncus murinus*); MJN Cl05–11 (EF641804, EF641806) from the Ussuri white-toothed shrew (*Crocidura lasiura*); CBN CBN-3 (EF543524, EF543525) from the Chinese mole shrew (*Anourosorex squamipes*); ARR MSB73418 (EF650086, EF619961) from the masked shrew (*Sorex cinereus*); JMS MSB144475 (FJ593499, FJ593501) from the dusky shrew (*Sorex monticolus*); SWS mp70 (EF636024, EF636026) from the Eurasian common shrew (*Sorex araneus*); RPL MSB89863 (FJ790772, EF540771) from the northern short-tailed shrew (*Blarina brevicauda*); ASA N10 (EU929072, EU929078) from the Japanese shrew mole (*Urotrichus talpoides*); and OXB Ng1453 (FJ539166, FJ593497) from the American shrew mole (*Neurotrichus gibbsii*). GenBank accession numbers are FJ539168 and FJ593498 for the NVA full-length S and L segments, respectively. The numbers at each node are posterior node probabilities based on 30,000 trees: two replicate Markov Chain Monte Carlo runs consisting of four chains of two million generations each sampled every 100 generations with a burn-in of 5,000 (25%). The scale bar indicates nucleotide substitutions per site.

**Figure 5:**
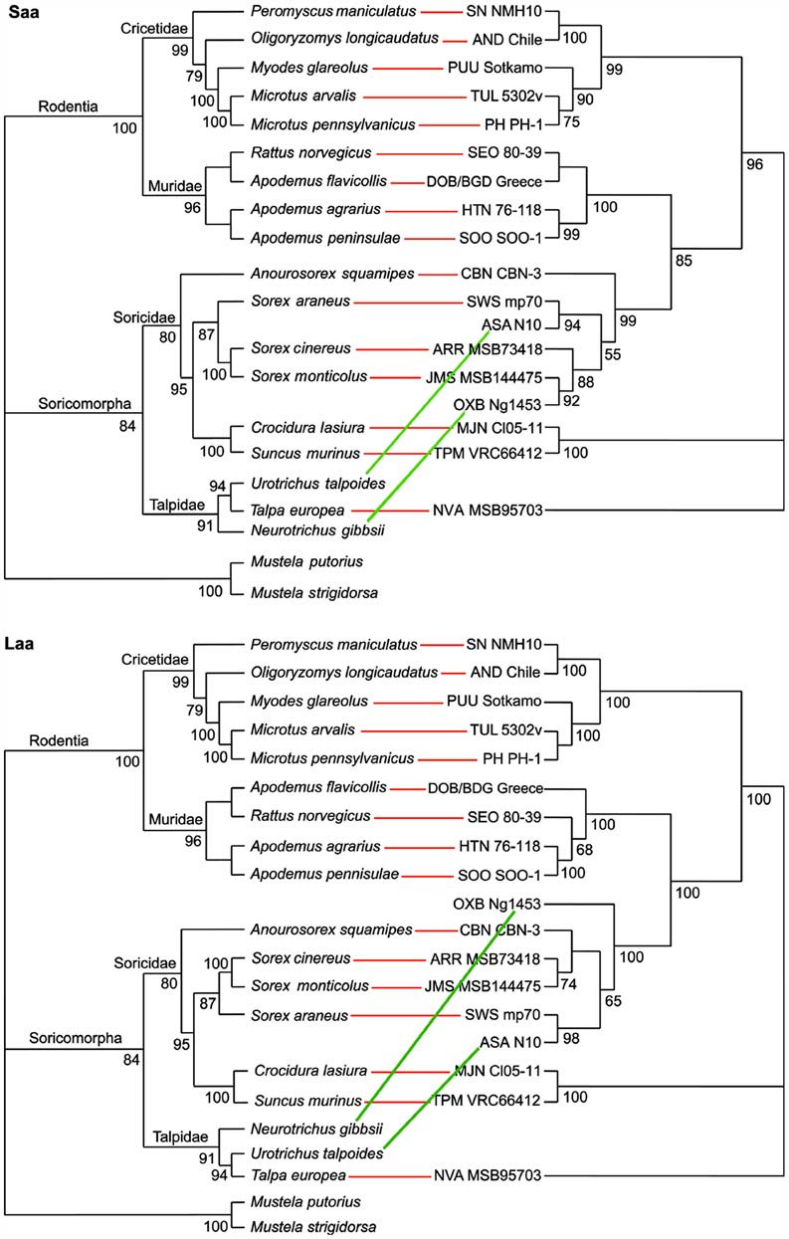
Tanglegrams, generated by TreeMap 2.0b, using consensus ML topologies based on the amino acid sequences of the nucleocapsid (N) protein (labeled Saa) and viral RNA-dependent RNA-polymerase (L protein) (labeled Laa) segments of NVA MSB95703 and representative rodent-, shrew- and mole-borne hantaviruses and cytochrome *b* mtDNA sequences of the respective reservoir host species. Node support was derived from 100 ML bootstrap replicates executed on the RAxML web server. Virus and host names are provided in the legend to Figure 4. Concordance of host and hantavirus cladograms was high (red line), except for two shrew mole-associated hantaviruses (ASA and OXB) which showed evidence of host switching (green line).

To clarify the phylogenetic relationship between NVAV and other hantaviruses, the entire coding regions of the S and L segments were analyzed by ML and Bayesian methods (Figure 4). Previously characterized rodent-associated hantaviruses segregated into three major clades according to their rodent host Subfamily (Murinae, Arvicolinae and Neotominae/Sigmodontinae) that were paraphyletic with a soricomorph-borne hantavirus clade. Hantaviruses harbored by soricomorphs were divided into two phylogenetic lineages (Figures 4 and 5): one lineage, which was paraphyletic with rodent-borne hantaviruses, included mainly soricine shrew-borne hantaviruses, with two notable exceptions of hantaviruses identified in shrew moles (ASAV and OXBV); the other group, which included crocidurine shrew-associated hantaviruses (TPMV and MJNV), was phylogenetically far removed from both soricine shrew- and rodent-associated hantaviruses. NVAV was very distantly related to all other hantaviruses, forming a separate branch, strongly supported by bootstrap analysis (0.9 or more), in both the S- and L-genomic segment-based phylogenetic trees (Figure 4). Highly congruent topologies (depicted as cladograms) were also found for the deduced amino acid sequences of the S and L segment-encoded proteins of NVAV and other hantaviruses (Figure 5). Moreover, tanglegrams, generated by TreeMap 2.0b, showed a high degree of concordance between hantaviruses and their respective reservoir host species (Figure 5), with the notable exception of ASAV and OXBV (harbored by shrew moles in Asia and North America, respectively), consistent with host switching. That is, the talpid-borne hantaviruses ASAV and OXBV were not phylogenetically associated with NVAV, but were more closely aligned to soricine shrew-borne hantaviruses and shared a common ancestor node.

### mtDNA Sequencing and Host Phylogeny

**Figure 6:**
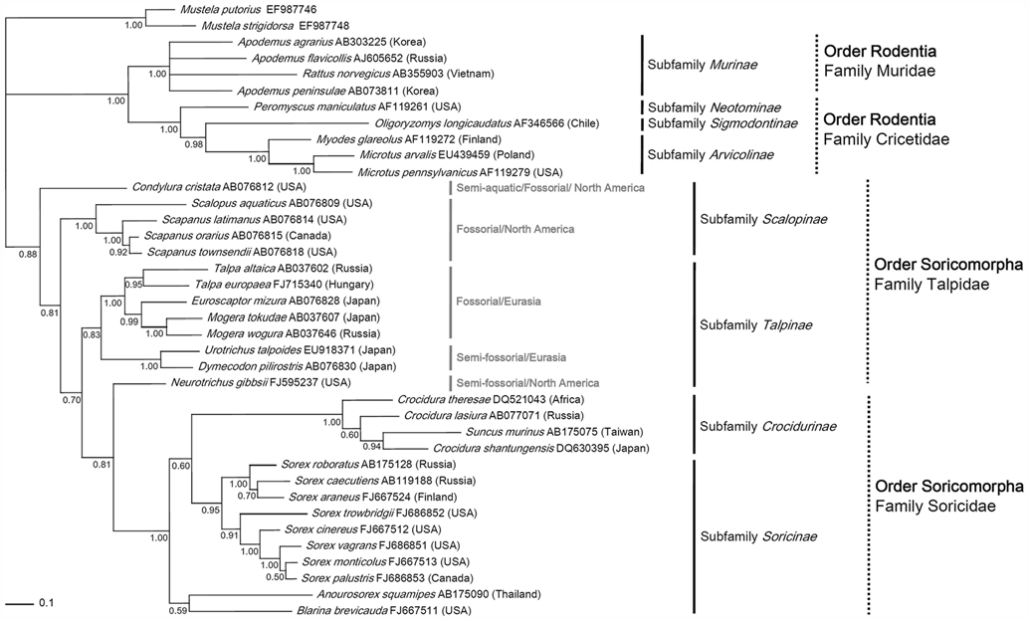
Maximum-likelihood phylogenetic trees, based on the 1,140-nucleotide cytochrome *b* region of mitochondrial DNA sequences of mammals in the Orders Rodentia and Soricomorpha. The phylogenetic position of the hantavirus-positive *Talpa europaea* (FJ715340) is shown in relationship to other *Talpa europaea* cytochrome *b* sequences from GenBank (AB037601, AB076829), as well as to other members of the Family Talpidae, as well as rodents and shrews. *Mustela putorius* (EF987746) and *Mustela strigidorsa* (EF987748) were used for the outgroup. The GenBank number and the country where each animal was captured are provided. Numbers at nodes are posterior node probabilities based on 30,000 trees: two replicate Markov Chain Monte Carlo runs consisting of four chains of two million generations each sampled every 100 generations with a burn-in of 5,000 (25%). The scale bar indicates nucleotide substitutions per site.

The identity of the European common mole, in which a novel hantavirus genome was detected, was verified by sequence analysis of the full-length 1,140-nucleotide cytochrome *b* gene. Phylogenetic analysis of the mtDNA cytochrome *b* gene confirmed that rodents, shrews and moles formed distinct lineages, and shrew and mole species formed a well-supported cluster distinct from that of rodent species (Figure 6). Moreover, within the cluster comprised of the Family Talpidae, *Talpa europaea* was more closely related to other fossorial moles from Eurasia (*Euroscaptor* and *Mogera*) than to fossorial moles from North America (*Scalopus* and *Scapanus*). Thus, segregation of talpids by both geography and life style was clearly evident.

## Discussion

### A Newly Identified Talpid-Borne Hantavirus

Previous reports of hantavirus antigens, as detected by enzyme immunoassay and immunofluorescence techniques, in tissues of the European common mole (Family Talpidae, Subfamily Talpinae) captured in Belgium [11] and Russia [12], [13], have now been corroborated by the molecular identification of a highly divergent hantavirus. NVAV, the first example of a hantavirus harbored by a true mole species, joins ASAV [27] and OXBV [28], found in the Japanese shrew mole and American shrew mole, respectively, in providing incontrovertible evidence that talpids, like soricids and rodents, serve as hosts of hantaviruses. Moreover, as supported by sequence and phylogenetic analyses, NVAV is the most genetically divergent hantavirus identified to date. Thus, an emerging conceptual framework posits a long-standing and well-established co-existence of hantaviruses in soricomorphs. Across this shared history of mammal and microparasite, examples of both co-speciation (arvicoline rodents *Myodes glareolus*, *Microtus arvalis* and *Microtus pennsylvanicus* with the viruses PUUV, TULV and PHV, respectively) and host switching (shrew moles *Neurotrichus gibbsii* and *Urotrichus talpoides* with the viruses OXBV and ASAV, respectively) are now suggested based on comparison of host and microparasite phylogenies [28]. As additional soricomorph-borne hantaviruses are identified, a more robust assessment of the relative role of these two processes in this host/parasite system will be possible. Accordingly, ancestral soricomorphs may have served as the original mammalian hosts of hantaviruses. In this hypothetical scenario, the primeval hantaviruses might have arisen from insect-borne viruses, in keeping with other genera of the Family Bunyaviridae, all of which have insect or arthropod vectors.

The Order Soricomorpha (formerly Insectivora) is comprised of five families: Soricidae (shrews), Talpidae (moles), Solenodontidae (solenodons), Erinaceidae (hedgehogs and gymnures) and Nesophontidae (Nesophontes). Although shrews and moles are occasionally mistaken as rodents, they belong to a different taxonomic Order, have a separate evolutionary history and distinctly dissimilar life histories [52], [53], such as age at maturity, litter size and maximum lifespan. At the same time, soricomorphs and rodents share ecological communities and certain physical characteristics, such as small body size [52]–[54]. Their additional common feature of harboring hantaviruses prompts questions about shared modes of virus transmission, such as intra- and inter-specific wounding [55] and virus shedding in secretions [10] and excretions [8]. Although salivary gland and terminal colon tissue were unavailable for testing, the detection of NVAV sequences in kidney tissue raises the possibility of viruria, which has been demonstrated for rodent-borne hantaviruses. In addition, immunological mechanisms of hantavirus persistence in shrews and moles, as proposed for rodents [56]–[58], and the role of hantavirus infection in promoting survival or causing premature mortality of soricomorphs warrants careful study.

### Host Switching and Co-divergence

The widely accepted notion that hantaviruses have co-evolved with their reservoir hosts [6], [50], [59] has been questioned recently by Ramsden and colleagues, who concluded there was no evidence of co-divergence between hantaviruses and their hosts, as determined by analysis of co-phylogenetic reconciliation and by estimation of evolutionary rates and divergence times [29]. Instead, they contended that preferential host switching and local host-specific adaptation during the more recent past account for the apparent similarities between the phylogenies of hantaviruses and their mammalian reservoir hosts [29].

As evidenced by multiple rodent species serving as reservoir hosts for the same hantavirus, host-switching events have definitely occurred during the evolution of hantaviruses [26], [60]. For example, Vladivostok virus has been found in its natural arvicolid rodent host, the reed vole (*Microtus fortis*) [61], [62], as well as in an ancillary host, the tundra or root vole (*Microtus oeconomus*) [63]. Similarly, Khabarovsk virus is carried by the Maximowiczi vole (*Microtus maximowiczii*), as well as *Microtus fortis* [62], [63]. And Topografov virus, which is closely related to Khabarovsk virus, has become well established in a distantly related rodent host of a different genus, the Siberian lemming (*Lemmus sibiricus*) [26].

Based on phylogenetic analysis of mtDNA cytochrome *b* and nuclear gene sequences and comprehensive morphological analysis, the European common mole is closely related to other Old World fossorial moles [64]–[67]. Our mtDNA cytochrome *b* analysis also showed that mole species in the Family Talpidae clustered according to geographic region (Eurasia vs. America) and life style (e.g., fossorial vs. semi-fossorial). Thus, if mole-borne hantaviruses have co-evolved with their reservoir hosts, they should exhibit parallel topologies with their hosts. This appears to be true for NVAV in *Talpa europaea*. However, the phylogenetic trees of hantavirus genomes recently detected in shrew moles (ASAV in *Urotrichus talpoides* and OXBV in *Neurotrichus gibbsii*) show them grouping with hantaviruses harbored by soricine shrew species in the New World and Old World, respectively. We interpret this polyphyletic relationship as being more consistent with host switching between species that share ecological communities than with deep co-divergence. In this regard, ample opportunities exist for hantavirus spillover into sympatric and syntopic species, particularly during irruptions in rodent reservoir populations [12], [68]. However, while examples of spillover or host switching between rodent species of the same family or subfamily are not uncommon [24]–[26], such events between small mammals of separate taxonomic families appear to be rare, suggesting that cross-species transmission is not a random and haphazard event determined simply by physical proximity, as in the sharing of virus-contaminated nesting materials and via aerosolized salivary secretions [69], but instead is likely driven by specific host behaviors and/or long-term virus-host adaptations.

Although cross-species transmission has undoubtedly influenced the course of hantavirus evolution, such host-switching events alone do not satisfactorily explain the co-existence and distribution of genetically distinct hantaviruses among host species in two divergent taxonomic Orders of small mammals spanning across four continents. The issue is not whether the evolutionary history of hantaviruses is a direct consequence of either host switching *or* co-divergence. Rather, when viewed within the context of molecular phylogeny and zoogeography, the close association between distinct hantavirus clades and specific subfamilies of rodents, shrews and moles is likely the result of episodes of host/pathogen co-divergence through deep evolutionary time. That is, hantaviruses have likely co-diverged with some of their reservoir hosts during part of their evolutionary history.

### Search for Other Talpid-Borne Hantaviruses

The discovery of genetically distinct soricid- and talpid-borne hantaviruses raises the question of what mammalian clade was the primordial reservoir hosts of hantaviruses. The highly divergent hantavirus in the European common mole suggests that other talpid species also harbor hantaviruses. High on the list of suspected candidate hosts is the eastern mole (*Scalopus aquaticus*) (Subfamily Scalopinae), which is the New World fossorial equivalent of the Old World European common mole in terms of its widespread distribution in much of the eastern half of the United States [70]. However, failure to detect hantavirus RNA in the eastern mole or other mole species may simply mean that suitable primers were not employed. In this regard, previous attempts to detect hantavirus RNA in the same European common mole tissues, using shrew-borne hantavirus-specific primers, were repeatedly unsuccessful. The vast genetic diversity and divergence of hantaviruses would seem to necessitate the design of specific primers for amplification in distinct hosts. In an effort to bypass this time-consuming, hit-and-miss process, a microarray approach aimed at detecting new hantaviruses harbored by soricomorphs is being explored. Also, the failure to amplify and sequence the NVAV M segment is reminiscent of the inability to sequence the TPMV M segment for more than a decade. Next-generation *de novo* sequencing technology may be necessary to obtain the complete NVAV genome.

### Secondary Structures of N Proteins

Remarkably little variation was found in the predicted structure of the N protein of NVAV and that of other hantaviruses [71], [72], despite the low to moderate level of amino acid sequence similarity. Therefore, the observed level of sequence variation should have little effect on the principal functions of the N protein, but differences and associated alterations in the secondary and tertiary structures can be expected to manifest themselves in the modulation of these functions. The well-conserved structure between homologous protein domains was compatible with a bi-lobed architecture in which the polypeptide chain folds into two large multi-helical domains connected together by a hinge-like β-sheet joint. The bi-lobed structure would allow the protein to clamp around the RNA in a clamshell-like fashion observed for various RNA-binding proteins. The NVAV N protein showed greatest homology with other hantaviruses at residues 175 to 217, which is known as the RNA-binding domain, and contained amino acids, E192, Y206, and S217, essential for RNA interaction [73], [74]. However, the RNA-binding site in NVAV may be markedly different in its specificity from most N proteins in the set due to an extra intervening α-helix in the RNA-binding β-sheet region. The specificity of protein associations may also be altered in NVAV and a few other hantaviruses. The coiled-coil structure in the first main α-helical domain is thought to be involved in the oligomerization of N proteins, as suggested previously for Tula virus [75]. The observed absence of the third α-helix in NVAV, OXBV and TPMV, as well as the segmentation of the second α-helical structure in HTNV, SOOV, SEOV and TPMV, may profoundly affect their protein-protein interactions.

While the overall structure of the N proteins appeared to be preserved during hantavirus evolution, more variability was seen in the number of constituent β-strands and in the adjoining connecting elements and smaller helices. The larger helical content in the second main domain of the NVAV N protein is indicative of decreased flexibility with a possible parallel change in the domain interfaces or RNA-binding sites. The central β-sheet region, which formed part of the RNA-binding site, was smaller in NVAV than in most other N proteins. However, the connecting and neighboring loops were longer, which may compensate for the diminished flexibility of the second main domain. The conformation of the central β-rich region may play an important role in RNA binding by modulating the interaction and presumptive binding surface between the two main domains, which could convey evolutionary advantages and virus specificity.

### Pathogenicity of Talpid-Borne Hantaviruses

While major gaps still remain in our understanding about the virus-host interactions in HFRS and HCPS, as well as the genetic determinants of tissue targeting and pathogenicity, our knowledge about the pathogenic potential of hantaviruses harbored by shrews and moles is nonexistent. For one, whether or not these soricomorph-associated hantaviruses infect humans and the types of diseases or syndromes they cause are completely unknown. However, because rodent-borne hantaviruses are capable of causing diseases as clinically disparate as HFRS and HCPS, shrew- or mole-associated hantaviruses might similarly cause a spectrum of diseases. That said, it might be argued that the risk of infection with soricid- or talpid-borne hantaviruses would be negligible, because human exposure to shrews and moles and to their presumably infectious secretions or excretions is extremely rare. This generalization is not altogether valid, because certain species of shrews and moles occasionally reside near or within human habitation and may be brought into homes by domestic cats. Thus, physicians should be vigilant for febrile diseases and other unusual syndromes occurring among individuals who report contact with shrews or moles. As an initial attempt to ascertain if NVAV, ASAV and OXBV are capable of infecting humans, recombinant N proteins are being prepared for use in enzyme immunoassays and Luminex-based tests to analyze sera from patients with febrile illnesses of unknown etiology, as well as sera from individuals, such as mammalogists and forestry workers, who have occupational exposures to soricomorphs.

## Supporting Information

Data S1 Original Table 2 Oligonucleotide primers
(0.05 MB PDF)
