# Supplementary material for: Evolutionary Insights from a Genetically Divergent Hantavirus Harbored by the European Common Mole (Talpa europaea)
Source: PLoS One. 2009 Jul 7;4(7):e6149. doi: 10.1371/journal.pone.0006149 (PMC2702001; doi:10.1371/journal.pone.0006149)
Supplement: Data S1 — Original Table 2 Oligonucleotide primers (0.05 MB PDF) [file pone.0006149.s001.pdf]

Table 2. Oligonucleotide primers for the amplification of the S- and L-genomic segments of NVAV.

Segment	Primer	Sequence (5′→3′)	Length	Polarity
S	OSM55	TAG TAG TAG ACT CC	14	+
	Han-S604F	GCH GAD GAR HTN ACA CCN GG	20	+
	Tal-S696F	TGA TNA GYC CTG TNA TGG GAG T	22	+
	Tal-S710F	ATG GGA GTG ATA GGC TTT CA	20	+
	Tal-S510R	ACC CGA TTA CCC TTA TGC TCT T	22	-
	Tal-S623R	CCD GGH GTN AGT TCW TCT GC	20	-
	Han-S974R	TCN GGN GCH CHN GCA AAN AHC CA	23	-
	HTN-S6	AGC TCN GGA TCC ATN TCA TC	20	-
	Han-S3R	TAG TAG TAN NCT CCY TRW ACA	21	-
	Han-S3R1	TAG TAG TAN NCT CCN	15	-
L	OSM55	TAG TAG TAG ACT CC	14	+
	IJV L181F	ATG AGA TGA TAA ARC ATG A	19	+
	Han-L1880F	CAR AAR ATG AAR NTN TGT GC	20	+
	Han-L2520F	ATN WGH YTD AAR GGN ATG TCN GG	23	+
	Tal-L2855F	GAA AGG GCA TTN MGA TGG GCN TCA GG	26	+
	Tal-L2928F	GNA AAY TNA TGT ATG TNA GTG C	22	+
	Tal-L3449F	GAN ATG TGG AAR NSN ATG TTY AA	23	+
	Tal-L4252F	TTY AGN ATG CAR GCN AAA CAG CA	23	+
	Tal-L4420F	TCA AAR GAR TWT GCA TGG AG	20	+
	Tal-L4552F	GTN ATA GSN TAT AGR TTT GC	20	+

Te-L4932F	TGT ATW GAA GTW TGG AGN TGG K	22	+
Tal-L5060F	TTG AYC CNG AAA TNC ART GT	20	+
Tal-L5125F	TNC ARA TGC ANG CAN ATA G	19	+
Te-L6409F	CCA GAG GAG TTA ATA CCT GA	20	+
Te-L340R	ATT TTR TAR TTR TCW GGT GT	20	-
Tal-L550R	ATT YCT YCT ACT NGG CCA	18	-
Tal-L1950R	GAA TAT TGA GAT GTG CAA GCA GG	23	-
Tal-L2145R	GAA GGR TAN AYN CCA CTD GCA CC	23	-
Tal-L2565R	ARA TGN CCA TCT TCT TGT A	19	-
Tal-L2715R	CCT CTA TCW GCT TCT GTN CKT TG	23	-
Han-L2970R	CCN GGN GAC CAY TTN GTD GCA TC	23	-
Han-L3470R	TTR AAC ATN SNY TTC CAC ATH TC	23	-
Han-L3588R	GGN ATH GAN ACN GCA CAN CCY TCA AA	26	-
Han-L5178R	CAA TAN GCA TCA TAY TGY TTN CC	23	-
Han-L3R	TAG TAG TAK GCT CCG NRR	18	-
Han-L3R-BamHI	TAT GGA TCC TAG TAG TAK GCT CCG NR	26	-
